# The demographic history of house mice (*Mus musculus domesticus*) in eastern North America

**DOI:** 10.1093/g3journal/jkac332

**Published:** 2022-12-21

**Authors:** Kennedy D Agwamba, Michael W Nachman

**Affiliations:** Center for Computational Biology, Museum of Vertebrate Zoology, University of California, Berkeley, Berkeley, CA 94720, USA; Center for Computational Biology, Museum of Vertebrate Zoology, University of California, Berkeley, Berkeley, CA 94720, USA

**Keywords:** demographic inference, population genetics, *Mus musculus domesticus*

## Abstract

The Western European house mouse (*Mus musculus domesticus*) is a widespread human commensal that has recently been introduced to North America. Its introduction to the Americas is thought to have resulted from the transatlantic movements of Europeans that began in the early 16th century. To study the details of this colonization history, we examine population structure, explore relevant demographic models, and infer the timing of divergence among house mouse populations in the eastern United States using published exome sequences from five North American populations and two European populations. For North American populations of house mice, levels of nucleotide variation were lower, and low-frequency alleles were less common than for European populations. These patterns provide evidence of a mild bottleneck associated with the movement of house mice into North America. Several analyses revealed that one North American population is genetically admixed, which indicates at least two source populations from Europe were independently introduced to eastern North America. Estimated divergence times between North American and German populations ranged between ∼1,000 and 7,000 years ago and overlapped with the estimated divergence time between populations from Germany and France. Demographic models comparing different North American populations revealed that these populations diverged from each other mostly within the last 500 years, consistent with the timing of the arrival of Western European settlers to North America. Together, these results support a recent introduction of Western European house mice to eastern North America, highlighting the effects of human migration and colonization on the spread of an invasive human commensal.

## Introduction

Untangling the invasion histories of human commensals helps to understand the impact of human migration on global ecosystems. Rats, pigeons, sparrows, zebra mussels, and fruit flies are a few of the many invasive species that humans have accidentally or intentionally helped disperse around the world (Robbins 1973; Astanei *et al.* 2005; Stringham *et al.* 2012; Jones *et al.* 2013; Puckett *et al.* 2016; Mallez and McCartney 2018; Arguello *et al.* 2019; Cucchi *et al.* 2020). The presence of these species often dramatically alters ecosystems, affecting the evolutionary trajectories of the organisms that interact with them and their shared ecological resources (Doherty *et al.* 2016; Early *et al.* 2016; Dueñas *et al.* 2018).

One of the most successful invasive species in recent history is the house mouse, *Mus musculus* (Pimentel *et al.* 2000; Genovesi *et al.* 2012). Originating in Asia on the Indian subcontinent, *Mus musculus* split into three parapatrically distributed subspecies, *Mus musculus castaneus, Mus musculus musculus*, and *Mus musculus domesticus*. *M. m. castaneus* is now found primarily in Southeast Asia. *M. m. musculus* is currently distributed throughout Northern Asia and Eastern Europe. *M. m. domesticus* was originally distributed across the Middle East, North Africa, and Western Europe, and has since been introduced worldwide in association with humans (Geraldes *et al.* 2008; Didion and de Villena 2013; Suzuki et al 2013; Phifer-Rixey and Nachman 2015; Morgan *et al.* 2022).

Commensalism between humans and house mice dates to roughly 12,000–15,000 years ago (Cucchi *et al.* 2005; Weissbrod *et al.* 2017). As humans developed farming practices and adopted more sedentary lifestyles, house mice were able to adapt to a variety of climates and live in close association with humans, taking advantage of stored grain as a readily accessible food source. From their origins in southwest Asia, zooarchaeological surveys have identified *M*. *m. domesticus* in the Near East and Eastern Mediterranean within the Neolithic approximately 10,000 years ago, and within the Western Mediterranean and Western Europe during the Iron Age around 3,000 years ago (Cucchi *et al.* 2005, 2020).

Only within the past 1,000 years have house mice migrated out of Western Europe (Rosevear 1969; Gabriel *et al.* 2010; Jones *et al.* 2013; Phifer-Rixey and Nachman 2015). House mice were spread throughout the North Atlantic as passengers of Viking ships during the 10th century, with *M. m. domesticus* having reached Iceland, Greenland, and possibly Newfoundland and the Azores, and *M. m. musculus* having more recently colonized Greenland (Searle *et al.* 2009; Jones *et al.* 2012; Gabriel *et al.* 2015). House mice from Western Europe likely similarly invaded the Americas as the unintended passengers of human migrants. The late 15th and early 16th centuries are known to have marked the beginning of significant interactions between the eastern and western hemispheres. The widespread exchange of organisms between the hemispheres played a tremendous role in shaping global biodiversity (Crosby 1972; Knight and Liss 1991; McCusker 2006; Jones *et al.* 2013). This event likely facilitated the establishment of *M. m. domesticus* populations in the Americas.

However, the demographic details of this recent colonization history have not been explored. In particular, the colonization of new areas may result in founder effects, the loss of genetic variation associated with population bottlenecks. Reductions in genetic variation in North American populations relative to source populations have been documented for diverse species including starlings (Cabe 1998), horses (Luís *et al.* 2006), and fruit flies (Arguello *et al.* 2019). In contrast, multiple colonization events from different source populations may result in admixture within the founding populations, leading to increased levels of genetic variation. Such a pattern is seen in brown rats which appear to have colonized North America from Asia multiple times (Puckett *et al.* 2016). It is not known whether house mouse populations in North America experienced founder effects and associated reductions in levels of genetic variation or admixture and associated increases in genetic variation. Finally, the timing of the colonization of North America by house mice has not been carefully studied. It remains unclear whether the earliest Europeans brought house mice with them, or whether house mouse populations were not established until much later, for example, during the industrial revolution when shipping between Europe and North America increased substantially.

To study the demographic history of house mice in eastern North America, we analyzed whole-exome data of mice from five populations in North America and two populations in Europe, focusing on three primary questions. (1) Was the colonization of eastern North America associated with a population bottleneck? (2) Do North American populations show mixed ancestry, as would be expected if multiple colonization events occurred from different source populations? (3) When did house mice begin to colonize North America? We discovered reduced genetic diversity and fewer rare alleles in North America than in Europe, consistent with a bottleneck. One population of house mice from Florida showed a strong signature of admixture, consistent with multiple introductions of mice to this region. Demographic modeling revealed recent split times between eastern North American populations, consistent with the timing of human migrants from Europe. These results highlight the close association between the movement of mice and humans and provide perspective into one of the world's most successful invasive species.

## Materials and methods

### Sample collection and sequencing dataset

**Fig. 1.**
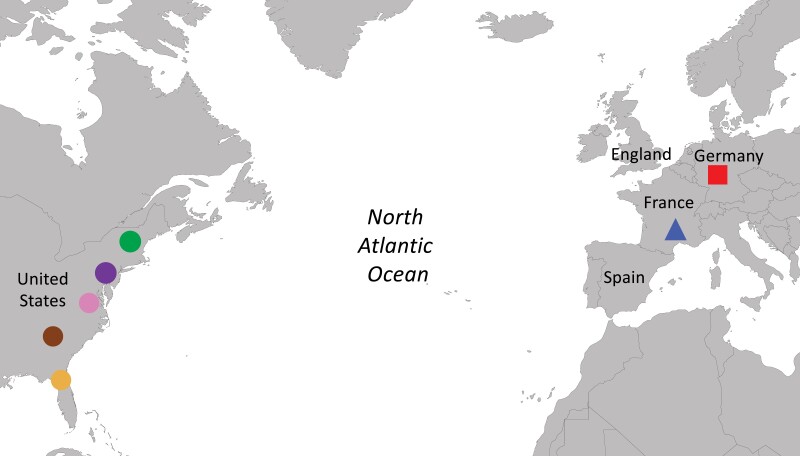
Locations of house mouse samples. Map showing locations of sampled mice from five populations in the eastern US (circles) from North to South as follows: New Hampshire/Vermont, Pennsylvania, Virginia, Georgia, and Florida, and from Germany (square), and France (triangle).

We compiled exome sequences of 66 wild house mice from published data representing five populations from eastern North America (Phifer-Rixey *et al.* 2018) and two populations from Europe (Harr *et al.* 2016; Fig. 1). The North American samples consisted of 50 mice from five localities along a latitudinal transect: New Hampshire/Vermont (NHVT); Pennsylvania (PA); Virginia (VA); Georgia (GA); Florida (FL). In brief, DNA was extracted from samples of mice collected from North America and Western Europe via DNAeasy kits (Qiagen, Hilden, Germany) or salt extractions. NimbleGen probes were used to capture exomes which were then sequenced at an average depth of ∼15×, as described by Phifer-Rixey *et al.* (2018). Sixteen *M. m. domesticus* samples from two European populations (Cologne-Bonn, Germany and Massif Central, France) and eight samples of *M. spretus* (Spain) were obtained via the European Nucleotide Archive (ENA: PRJEB9450); these were sequenced with an average depth of ∼20× (Harr *et al.* 2016). Reads from North America and Western Europe were mapped against the mouse GRCm38/mm10 reference genome using novoalign and bwa-mem, respectively (Mu *et al.* 2012; Li 2013). In all cases, wild mice were caught more than 500 m from one another to avoid sampling related individuals.

Exomes were extracted from whole genome samples using *bedtools* (Quinlan and Hall 2010). The *samtools* “mpileup” command was used to call variants (Li 2011), filtering away sites with greater than 50% missing data and/or quality scores less than 30. VCF files were then compressed, indexed, and merged at intersecting positions using *vcftools* (Danecek et al 2011). After this stage of filtering, 955,312 single nucleotide polymorphisms (SNPs) were retained. Further filtering was done using PLINK to remove Hardy–Weinberg outliers and prune for linkage disequilibrium using a window size of 50 kb and a *r*^2^ threshold of 0.5. After LD pruning, 395,879 SNP were passed on for downstream analysis. The final VCF file is available on Dryad (https://doi.org/10.6078/D1CX2R).

### Population structure

We estimated phylogenetic relationships among individuals using RAxML (Stamatakis 2014). A maximum likelihood phylogeny was constructed using concatenated SNPs representing all populations under the GTRGAMMA model with rapid bootstrapping, and using *M. spretus* as an outgroup.

We conducted principal component analysis (PCA) using PLINK to examine population structure (Purcell *et al.* 2007). The eigenvectors and eigenvalues associated with the first 20 principal components were derived using PLINK and the first two principal components were plotted in R (R Core Team 2021). ADMIXTURE was used to infer shared ancestral tracts between sample subpopulations represented in the genomic datasets (Alexander *et al.* 2009). PLINK was used to generate the input bed file for ADMIXTURE from a filtered VCF file containing the seven *M. m. domesticus* populations included in the PCA and one population of *M. spretus*. *M. spretus* was included in all admixture analyses since introgression between *M. musculus* and *M. spretus* is known to occur in Europe (Song *et al*. 2011; Liu *et al.* 2015; Banker *et al.* 2022).

*F*_st_ was used to quantify the extent of genetic divergence observed between populations in relation to the amount of variation observed within populations. Cockerman–Weir's weighted *F*_st_ was computed using vcftools “—weir-fst-pop” flag on filtered exomic SNPs from French, German, and eastern North American samples of *M. m. domesticus*.

Nucleotide diversity (*π*) and Watterson's theta (*θ*) are estimators of the neutral mutation parameter, 4*N*_e_*µ*, where *N_e_* is the effective population size and *µ* is the neutral mutation rate. Tajima's D (Tajima 1989) is the normalized difference between *π* and *θ*. These estimators, together with the distribution of allele frequencies for all SNPs (the site frequency spectrum), summarize present levels of variation and can be used to illuminate past demographic processes. We estimated *π* and *θ* using ANGSD (Korneliussen *et al.* 2014). ANGSD generates per-site sample allele frequency likelihoods, as well as the folded site frequency spectrum using realSFS. Log posterior probabilities were computed to derive *π*, *θ*, and Tajima's D from the folded SFS.

### Demographic inference and analysis

We used *f*_3_ statistics to explore the possibility that multiple source populations contributed to present populations of house mice in eastern North America. This statistic is based on a three-population comparison, and negative values of the test statistic provide evidence of admixture. We used the five North American and two European populations of *M. musculus* as well as the population of *M. spretus*, and we calculated *f*_3_ for each of the 56 possible three-way comparisons among these eight samples. *f*_3_ statistics were computed using the “threepop” command in TreeMix (Reich *et al.* 2009; Patterson *et al*. 2012; Pickrell and Pritchard 2012). We used Admixture-induced Linkage Disequilibrium for Evolutionary Relationships (ALDER) to generate test admixture statistics and to infer the approximate time of mixture (Loh *et al.* 2013). Reported parameter results correspond to simultaneously computed two-reference and one-reference models.

We used the Diffusion Approximation for Demographic Inference (*dadi*, Gutenkunst *et al.* 2009) and Genetic Algorithm for Demographic Model Analysis (GADMA, Noskova *et al.* 2020) to explore the demographic history of *M. m. domesticus* populations. We estimated two-dimensional SFS from SNP allele frequencies using ANGSD, requiring a minimum mapping quality of 30, base quality score of 20, and the removal of poorly mapped, duplicate, non-unique, or unpaired reads. To mitigate the effects of selection, we restricted demographic analyses to synonymous SNPs, predicted using variant effects predictor, when generating per-site allele frequencies (McLaren *et al.* 2016).

Folded joint SFS were prepared as input for *dadi* using realSFS with the “dadi” flag option. We explored a variety of demographic models taking the general form of an ancestral population that gives rise to two descendant populations, with and without migration between them. Parameters in the models included divergence time, population sizes, and migration rates. *dadi* was used to estimate these parameters in a likelihood framework. We used GADMA to carry out a global heuristic search of parameter space via the genetic algorithm (GA) to infer the best fit demographic model. Parameter values in *dadi* are scaled to *θ* = 4*N*_*anc*_*μ*, the neutral mutation parameter as a function of the ancestral effective population size and mutation rate per generation. We used this relationship to convert the output to standard units (e.g. *N*_e_, *t* in years, and *m*) using the per-site mutation rate *μ* = 4 × 10^−9^ and a generation time of 1 year (Geraldes *et al.* 2008). To generate confidence intervals for the parameter estimates, we divided each dataset into non-overlapping 10 kb sections and simulated pseudo-replicate datasets by sampling with replacement for bootstrapping under the parameters that generated the maximum likelihood for the best fitting model. The standard deviations for all parameters were derived from uncertainty analysis using the Godambe Information Matrix for composite likelihoods.

## Results

### Population structure

**Fig. 2.**
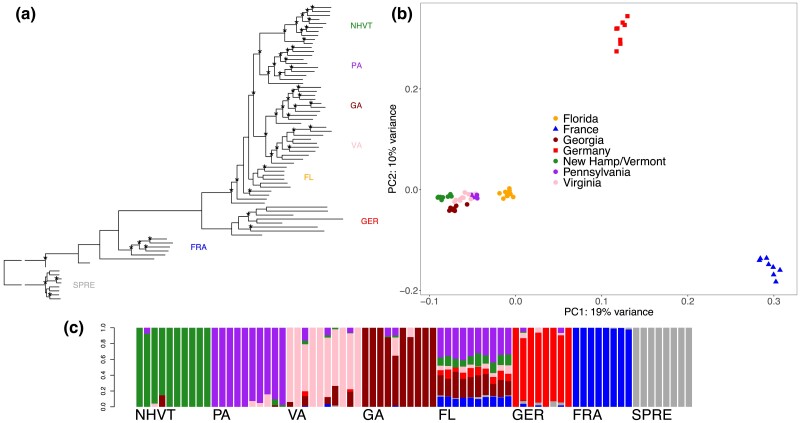
Analysis of population structure. a) Phylogenetic tree of *M. m. domesticus* constructed using RAxML with *M. spretus* as an outgroup. *M. m. domesticus* are from Florida (FL, *n* = 10), Georgia (GA, *n* = 10), Virginia (VA, *n* = 10), Pennsylvania (PA, *n* = 10), New Hampshire/Vermont (NHVT, *n* = 10), Germany (GER, *n* = 8), and France (FRA, *n* = 8). Asterisks indicate nodes with > 80% bootstrap support. b) Principal component analysis of 66 wild-caught *M. m. domesticus*. c) Inferred admixture proportions for *K* = 7 distinct ancestral subpopulations for eastern North American, German, and French *M. m. domesticus* populations, and an outgroup *M. spretus* population.

Phylogenetic reconstruction with RAxML was used to uncover the relationships among mice from Europe and eastern North America (Fig. 2a). Using *M. spretus* as an outgroup, mice from France were paraphyletic with respect to the remaining populations in the phylogeny. Mice from Germany formed a monophyletic group, and this was the sister group to mice from North America. Among the samples from North America, all populations except Florida formed monophyletic groups. Mice from Florida were paraphyletic with respect to the remaining North American populations.

Samples from Germany, France, and eastern North America formed separate clusters when plotted with the first two principal components which together accounted for 29% of the variation (Fig. 2b). Among the North American samples, individual localities largely formed distinct clusters, although the positions of these clusters did not correspond with the geographic distance between populations. The Florida population was unique in clustering farther from other North American samples.

To further examine the structure of populations, we used ADMIXTURE to infer shared ancestry proportions. When modeling for distinct ancestral clusters at *K* = 7 individuals from all populations except Florida showed little to no admixture (Fig. 2c). In contrast, all individuals from the Florida population showed high levels of admixture. Notable levels of admixture were inferred in the Florida population across all values of K (Supplementary Fig. 1).

The average *F*_st_ among populations in North America was 0.092 while *F*_st_ between the two European populations was 0.168, despite the fact that the European populations are separated by ∼1,000 km while the most distant North American populations are separated by ∼2,000 km. Pairwise *F*_st_ among all populations ranged from 0.041 to 0.281, with the lowest levels of differentiation seen between Florida and Pennsylvania and the greatest levels of differentiation seen between France and New Hampshire/Vermont (Table 1).

**Table 1.** Pairwise *F*_st_ comparisons between two western European population (France and Germany) and five eastern North American population samples.

	France	Germany	New Hampshire/Vermont	Pennsylvania	Virginia	Georgia
Germany	0.168					
New Hampshire/Vermont	0.281	0.202				
Pennsylvania	0.215	0.143	0.124			
Virginia	0.222	0.146	0.135	0.087		
Georgia	0.237	0.170	0.159	0.106	0.056	
Florida	0.161	0.101	0.096	0.041	0.054	0.063

#### Reduced genetic diversity and the distribution of allele frequencies suggest a bottleneck associated with the founding of *M. m. domesticus* populations in eastern North America

A reduction in genetic diversity in a derived population relative to the source population is expected following a recent population bottleneck. We estimated per-site nucleotide diversity (*π*) and Watterson's theta (*θ*) from allele frequencies computed in ANGSD. The populations from Germany and France had substantially higher levels of nucleotide diversity (average *π* = 0.28%) than any of the North American populations (average *π* = 0.17%; Table 2). Similarly, populations from Germany and France had a higher proportion of segregating sites (average *θ* = 0.26%) than any of the North American populations (average *θ* = 0.15%; Table 2).

**Table 2.** Summary of genetic diversity in sampled *M. m. domesticus* populations.

Population	Avg per-site Watterson's theta (%)	Avg per-site nucleotide diversity (%)	Tajima's *D*
France	0.269	0.289	0.438
Germany	0.252	0.271	0.440
New Hampshire/Vermont	0.121	0.140	0.754
Pennsylvania	0.149	0.169	0.649
Virginia	0.155	0.170	0.472
Georgia	0.133	0.151	0.641
Florida	0.188	0.205	0.453

Population bottlenecks are also expected to lead to a loss of rare alleles, thus skewing the shape of the allele frequency spectrum in comparison to source populations. The proportion of segregating sites was consistently lower than pairwise nucleotide diversity, leading to a positive Tajima's D for all populations. However, Tajima's D was consistently larger in populations from eastern North America than in European populations (Table 2), indicating a greater scarcity of rare alleles in the American populations. This pattern can also be seen in the folded site frequency spectra for the individual populations (Supplementary Fig. 2). In particular, the North American populations show more intermediate frequency alleles (relative to neutral expectations) compared to the European populations.

### Evidence of admixture in house mice from Florida

To test the hypothesis that some North American populations may have arisen from multiple introductions, we used three-population *f* statistics. We computed all 56 (8 choose 3) possible *f*_3_ statistics using the seven population samples of *M. m. domesticus* and the single population sample of *M. spretus*. Of these 56 tests, eight returned a negative value, all involving the Florida population, suggesting that this population is admixed (Table 3). Four comparisons suggest that allele frequencies in Florida are intermediate between those of populations from France and populations from the remaining four North American populations (Table 3). The remaining four comparisons suggest that allele frequencies in Florida are intermediate between those of *M. spretus* and the remaining four North American populations. While it is unclear whether the sampled populations are the direct contributors to the admixture observed in Florida, a negative *f*_3_ statistic implies that a phylogenetic tree for the given populations is a poor fit to the data without admixture along an internal branch (Supplementary Fig. 3). The inference of admixture in the Florida population is consistent with several other observations, including the fact that this population has the highest nucleotide diversity in North America (Table 2), the lowest levels of differentiation from the other sampled populations (Table 1), is phylogenetically paraphyletic with respect to the other North American populations (Fig. 2a), and appears admixed in other analyses (Fig. 2c).

**Table 3.** Summary and admixture statistics and inferred demographic parameters.

Population A (Target)	Population B	Population C	*F*_3_ statistic	s.e.	Z-score	Pop B 1-ref decay	Pop B 1-ref amp_exp	Pop B 1-ref z-score	Pop C 1-ref decay	Pop C 1-ref amp_exp	Pop C 1-ref z-score	2-ref decay	2-ref amp_exp	2-ref z-score	2-ref *P*-value (test status)
Florida	France	New Hampshire/Vermont	−5.62e−3	6.29e−4	−8.92869	102.08 ± 45.83	3.91e−4 ± 1.65e−4	2.23	87.25 ± 19.31	1.46e−4 ± 4.72e−5	3.08	154.42 ± 106.08	1.59e−3 ± 2.45e−3	0.65	0.52 (failure)
Florida	France	Pennsylvania	−1.66e−3	4.84e−4	−3.43814	102.08 ± 45.83	3.91e−4 ± 1.65e−4	2.23	2.00 ± inf	−3.66e−6 ± inf	0	16.49 ± 57.05	8.97e−5 ± 2.58e−4	0.29	0.77 (failure)
Florida	France	Virginia	−1.01e−3	5.06e−4	−1.98888	102.08 ± 45.83	3.91e−4 ± 1.65e−4	2.23	13.46 ± 28.09	1.66e−5 ± 1.96e−5	0.48	2.67 ± inf	6.76e−5 ± inf	0	1 (failure)
Florida	France	Georgia	−2.3e−3	5.33e−4	−4.31575	102.08 ± 45.83	3.91e−4 ± 1.65e−4	2.23	2.00 ± inf	−3.83e−6 ± inf	0	4.82 ± inf	5.25e−5 ± inf	0	1 (failure)
Florida	*M. spretus*	New Hampshire/Vermont	−8.57e−3	1.1e−3	−7.79405	2.00 ± inf	1.42e−3 ± inf	0	87.25 ± 19.31	1.46e−4 ± 4.72e−5	3.08	2.00 ± inf	1.01e−3 ± inf	0	1 (failure)
Florida	*M. spretus*	Pennsylvania	−6.14e−3	8.32e−4	−7.38383	2.00 ± inf	1.42e−3 ± inf	0	2.00 ± inf	−3.66e−6 ± inf	0	2.00 ± inf	1.26e−3 ± inf	0	1 (failure)
Florida	*M. spretus*	Virginia	−3.91e−3	8.51e−4	−4.59203	2.00 ± inf	1.42e−3 ± inf	0	13.46 ± 28.09	1.66e−5 ± 1.96e−5	0.48	2.00 ± inf	1.36e−3 ± inf	0	1 (failure)
Florida	*M. spretus*	Georgia	−4.36e−3	8.87e−4	−4.91237	2.00 ± inf	1.42e−3 ± inf	0	2.00 ± inf	−3.83e−6 ± inf	0	2.00 ± inf	1.05e−3 ± inf	0	1 (failure)

Remaining columns are inferred demographic parameters generated using ALDER.

Negative f3 statistic indicating admixture, standard error, and associated *z*-score were computed with TreeMix.

We further investigated the details of admixture in Florida using ALDER. ALDER estimates the dates of plausible admixture events while also providing a formal 2-reference weighted LD statistic to test if the two potential source populations are the probable contributors to the admixed population. This analysis returned no significant 2-reference weighted LD statistics using any two pairs of populations as the contributing populations for Florida's admixture (Table 3). We did, however, observe significant 1-reference weighted LD statistics using France and New Hampshire/Vermont (*P* = 0.012874 and *P* = 0.001035, respectively). From the decay rate, we estimated a time to admixture from France (or a related population) of 102.08 ± 45.83 years, assuming one generation per year. We estimated a time to admixture from New Hampshire/Vermont (or a related population) of 87.25 ± 19.31 years.

#### Demographic modeling suggests a recent introduction of *M. m. domesticus* to eastern North America

**Fig. 3.**
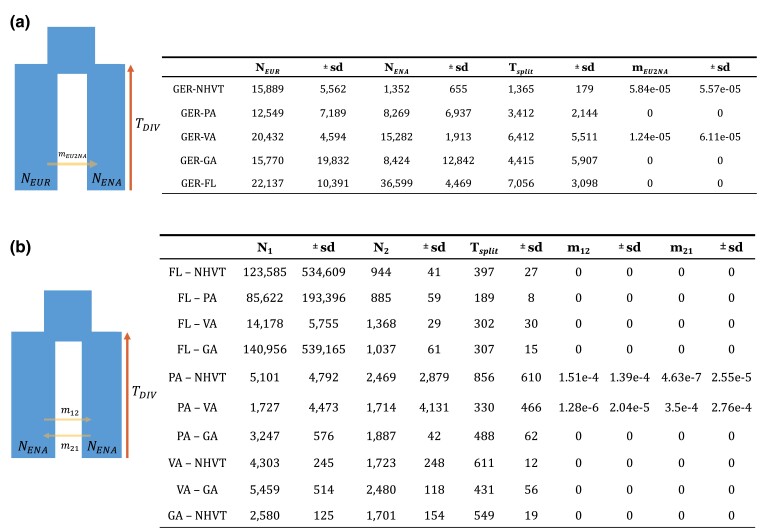
Two-population split modeling using Europe and eastern North American populations. a) The models depict a split occurring at time *T*_DIV_ years ago, leading to present-day populations of *N*_EUR_ and *N*_ENA_, allowing for continued unidirectional migration from Europe to eastern North America. b) The model is similar to a) but allowing for bidirectional migration, reported as parameters *m*_12_ and *m*_21_. Best fit demographic parameters inferred under these two divergence models are reported to the right.

We used *GADMA* to infer divergence times and effective population sizes under a variety of two-population models for all pairs of populations from Germany, France, and North America. First, we estimated demographic parameters between Germany and France. Next, we compared each North American population with each European population, constraining the inferred divergence time to the recent history during which house mice are known to have emerged from the Middle East (past 7,500 years). Finally, we compared North American populations with each other, again using a constraint on divergence time of 7,500 years. In all cases, we compared results under a simple divergence model without migration to a divergence model including migration (Fig. 3). Since these models are nested, we used a likelihood ratio test (LRT) to determine if the inclusion of migration improved the fit of the models. The standard deviations of the estimated parameters in these models were generally large (Fig. 3). Thus, the estimates of parameter values should not be taken as precise; nonetheless, several clear patterns emerged.

The estimated divergence time under a simple split model without migration comparing Germany and France was 2,584 ± 11 ya. This is consistent with zooarchaeological evidence which suggests that *M. m. domesticus* spread through western Europe during the Iron Age, which ended inside of 2.5 kya (Cucchi et al 2005; Jones *et al.* 2013). When migration was incorporated into the model, a similar divergence time was inferred (2,871 ± 1,444 ya) and the inferred migration rates were zero.

The estimated divergence times under two-population split models when comparing Germany and populations from North America were between 1 and 7 kya (Fig. 3a), within the range of the late Neolithic/Copper Age and late Iron Age, and overlapping in confidence interval with the divergence time between Germany and France. Inference of demographic parameters under two-population modeling for France and eastern North American populations failed to converge under the specified time constraints for divergence time. Nonetheless, the deep age estimates between German and North American populations predate the known arrival of European settlers to the Americas by thousands of years, suggesting that the source populations for North American mice were not from France or Germany.

In these models, the estimated effective population sizes for North American populations were generally smaller than those of the European populations (Fig. 3a), consistent with a bottleneck during the founding of most North American populations. The one exception to this pattern involved the Florida population, which revealed a higher effective population size when compared to the population from Germany. The higher estimated *N_e_* most likely reflects admixture in Florida, as documented above.

Finally, we considered models with pairs of North American populations. Under a simple split model without migration, inferred divergence times were mostly within the last 500 years, corresponding to the timing of human colonization (Fig. 3b). Likelihood ratio tests almost uniformly supported divergence models without migration. The one case where the LRT favored a model with migration was the Virginia–Pennsylvania comparison (*P* = 0.0143; Supplementary Table 1). In that case, the model with migration also suggested a divergence time of 300 years ago. Similarly, the LRT narrowly failed to reject a model without migration for New Hampshire–Vermont and Pennsylvania (*P* = 0.0566), and a model with migration suggested a divergence time (856 years ago) whose confidence interval substantially overlaps the divergence times estimated from other populations pairs (Fig. 3b; Supplementary Table 1). In sum, these models indicate that pairs of North American populations share ancestry within the time frame of European colonization of the Americas.

## Discussion

Here, we connect the invasion history of house mice in the eastern United States to the colonization of the Americas by European settlers which began in the 16th century. Previous genetic studies on the recent colonization history of house mice have mainly used mitochondrial DNA and microsatellites and have focused on the colonization of islands in the Atlantic including Iceland, Greenland, New Foundland, Gough Island, and the Azores (Jones *et al.* 2012; Gray *et al.* 2014; Gabriel *et al.* 2015). These studies revealed close associations between human and mouse colonization histories but did not address the connections between US and European house mice.

Using genome-wide data, we found that North American populations of house mice underwent a bottleneck compared to European populations. We discovered that one population in Florida shows evidence of considerable admixture, suggesting that house mice may have colonized parts of North America from distinct sources. Finally, we found that divergence times among North American populations of house mice correspond roughly to the timing of colonization by Europeans. Below we discuss each of these in turn.

Evidence for a bottleneck comes from several sources. First, levels of genetic variation are substantially lower in North American populations of house mice than in the European populations. For example, the average nucleotide diversity in North America (*π* = 0.17%) was only 60% of that seen in European populations (*π* = 0.28%). Second, Tajima's D was more positive in North American populations than in European populations, consistent with the loss of rare alleles. Finally, demographic modeling using *dadi* revealed smaller population sizes for most North American populations compared to the German populations (Fig. 3b), with the one exception being the population from Florida, as discussed below. In aggregate, these results indicate that the founding of North America was accompanied by a modest bottleneck.

From the 16th century onward, numerous ships from various parts of Western Europe landed on the shores of eastern North America. Many intentionally transported non-native species, such as horses, cattle, pigs, sheep, rock pigeons, house sparrows, and starlings (Robbins 1973; Cabe 1998; Giuffra *et al.* 2000; Luís *et al.* 2006; Ajmone-Marsan *et al.* 2010; Stringham *et al.* 2012; Bertolini *et al.* 2018). Several ships also unintentionally harbored invasive species, such as rats, mice, zebra mussels, and fruit flies (Duffy 1951; Astanei *et al.* 2005; Aplin *et al.* 2011; Jones *et al.* 2013; Puckett *et al.* 2016; Mallez and McCartney 2018; Arguello *et al.* 2019). In many cases, the resulting founder populations experienced modest bottlenecks through their introductions (Cabe 1998; Luís *et al.* 2006; Puckett *et al.* 2016; Bertolini *et al.* 2018; Arguello *et al.* 2019).

Evidence of multiple colonization events from distinct sources comes from observations of admixture in the Florida population (Fig. 2c; Table 3). Lack of significance among all 2-reference weighted LD statistics using ALDER suggests that the Florida population is not the direct result of admixture from any of the populations included in this study (Table 3). Florida has a complex colonization history involving Spanish and British rule (Hudson 1997; Glanville 2009), and it is possible that both groups of people introduced house mice to the region. The Spanish first explored Florida in 1513, when Juan Ponce de Leon recorded his exploration of the peninsula. The Spanish ruled over Florida until 1763, when the British overtook Spanish rule for 20 years, before the Spanish reestablished sovereignty over Florida in 1783, though many British and American settlements in Florida remained. The second Spanish occupation of Florida lasted until 1821, at which point, Florida became an organized colony of the United States (Knight and Liss 1991; Hudson 1997; McCusker 2006; Glanville 2009). Future studies with better sampling across Europe would help to identify the possible source populations contributing to admixture in Florida.

**Fig. 4.**
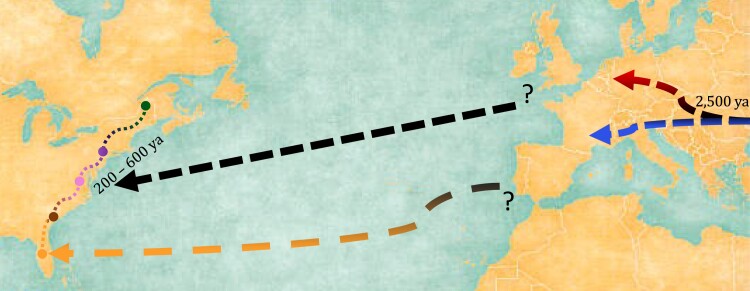
Recent progression of house mice from Western Europe to Eastern North America. Hypothesized colonization history of house mice in Eastern North America showing divergence times as inferred from demographic modeling. Black dashed arrow illustrates migration of house mice from Western Europe to eastern North America. Orange dashed arrow illustrates additional migration from Western Europe to Florida. Lines connecting North American populations illustrate possible spread across the Eastern coast of North America during founding period. Dashed arrows in Europe depict the ancestral split between European populations from Cologne-Bonn, Germany, and Massif Central, France. Colors match Figs. 1 & 2.

To ask whether divergence times of mouse populations reflect the timing of human settlements, we estimated divergence times between each pair of populations using *dadi*. Direct comparisons of eastern North American populations with one another largely yielded split times within the past 500 years, consistent with the known period of European colonization in the Americas. Inferred divergence times between Germany and eastern North America fell within the range of the late Neolithic to Iron Age period (5,000 BC–1,000 AD) (Cucchi *et al.* 2005, 2012, 2020). Similarly, the divergence time inferred between Germany and France was around 2.5 kya. Consistent with zooarchaeological evidence, the late bronze age to iron age are the time periods when house mice progressed toward western Europe from the Middle East. These observations suggest a scenario in which mice in eastern North America came from an unsampled population in Western Europe. The unsampled population likely diverged from the German and French populations sometime in the last few thousand years (Fig. 4).

Human colonization history suggests that source populations for North American mice are likely to have come from northern Europe (Knight and Liss 1991; McCusker 2006). Virginia is known to have included the first enduring British settlement of the Americas in Jamestown, beginning in 1607. Pennsylvania became a British colony in 1681. Prior to the British, the Dutch and Swedish had established colonies in Pennsylvania early in the 17th century. New Hampshire was also one of the original 13 colonies, with the first British settlements established around 1623. For most of pre-American colonial history, New York and New Hampshire had split claim over the territory that is now the state of Vermont. Georgia, named after King George II of Great Britain, became the last of the 13 colonies in 1752. Given the predominance of British rule through much of the recent colonization history of America, England is a reasonable source location for North American mice. Moreover, British migration through the eastern seaboard in establishing the 13 colonies may have facilitated the spread of house mice from initial sites of introduction.

Despite the strong British influence, the Spanish were the first Europeans to establish consistent transatlantic exchanges with North America and thereby had the earliest opportunities to introduce non-native organisms. A recent study by Morgan *et al.* (2022) used a SNP genotyping array to assess population structure across Europe and elsewhere and found that mice in Europe consist of a northern clade (including Germany, Denmark, Belgium, and Scotland) and several southern groups (including Greece, Italy, Portugal, and Spain). Their study did not include samples from England, but one interesting possibility is that mice in eastern North America derive primarily from England, but that mice in Florida may represent a mixture of mice from England and mice from southern Europe. This hypothesis could be tested by deep sampling of mice across Europe.

## Conclusion

We conclude that house mice in the eastern United States arrived and dispersed largely within the past 500 years. Their arrival was associated with a genetic bottleneck, as US populations harbor only 60% of the average genetic variation seen within European populations. Since the arrival of the initial source population, at least one other source population successfully mixed with an established population in Florida. Notably, Florida was also colonized by different Western European settlers within the past few centuries. The overall similarity in the estimated divergence times of North American populations of house mice with the known timing of human colonization of eastern North America suggests a very close association between the movements of humans and mice and suggests that the earliest European settlers may have brought mice with them.

## Supplementary Material

jkac332_Supplementary_Data

## Data Availability

All genomic data used in this study were from previously published studies (Harr *et al.* 2016; Phifer-Rixey *et al.* 2018), available under ENA PRJEB9450 and NCBI SRA PRJNA397150. A VCF file has been deposited in DRYAD (doi: https://doi.org/10.6078/D1CX2R). The authors affirm that all data necessary for confirming the conclusions of the article are present within the article, figures, and tables. Supplemental material available at G3 online.

## Notes

*Communicating editor: P. Ingvarsson*
