# Supplementary material for: The demographic history of house mice (Mus musculus domesticus) in eastern North America
Source: G3 (Bethesda). 2022 Dec 21;13(2):jkac332. doi: 10.1093/g3journal/jkac332 (PMC9911051; doi:10.1093/g3journal/jkac332)
Supplement: jkac332_Supplementary_Data [file jkac332_supplementary_data.zip › Supplementary_Table_1_G3-2022-403878.pdf]

	MLCL (w/o migration model)	MLCL (w/ migration model)	Adjusted D-statistic	p-value
GER-FRA	-7138	-7171	-0.4551	1.0000
GER-NHVT	-6821	-6783	0.2276	0.3167
GER-PENN	-5519	-5518	0.0609	0.4025
GER-VIRG	-5619	-5614	0.3273	0.2836
GER-GEOR	-6033	-6026	0.1484	0.3500
GER-FL	-4778	-4782	-0.6041	1.0000
FRA-NHVT	-8373	-8376	-0.0154	1.0000
FRA-PENN	-6862	-6859	0.0379	0.4228
FRA-VIRG	-7051	-7053	-0.0002	1.0000
FRA-GEOR	-7476	-7482	-0.0769	1.0000
FRA-FL	-5977	-5977	0.0009	0.4883
FL-NHVT	-3477	-3210	0.8440	0.1791
FL-PENN	-1847	-1592	1.9043	0.0838
FL-VIRG	-1979	-1861	0.8648	0.1762
FL-GRG	-2431	-2131	1.4598	0.1135
VIRG-NHVT	-3555	-3589	-1.2546	1.0000
VIRG-PENN	-2386	-2379	4.7886	0.0143
VIRG-GRG	-1886	-1708	2.2615	0.0663
PENN-NHVT	-3338	-3336	2.5081	0.0566
PENN-GRG	-2856	-2918	-4.1012	1.0000
GRG-NHVT	-3786	-3863	-12.9825	1.0000
