# Supplementary figures and images for: The demographic history of house mice (Mus musculus domesticus) in eastern North America
Source: G3 (Bethesda). 2022 Dec 21;13(2):jkac332. doi: 10.1093/g3journal/jkac332 (PMC9911051; doi:10.1093/g3journal/jkac332)

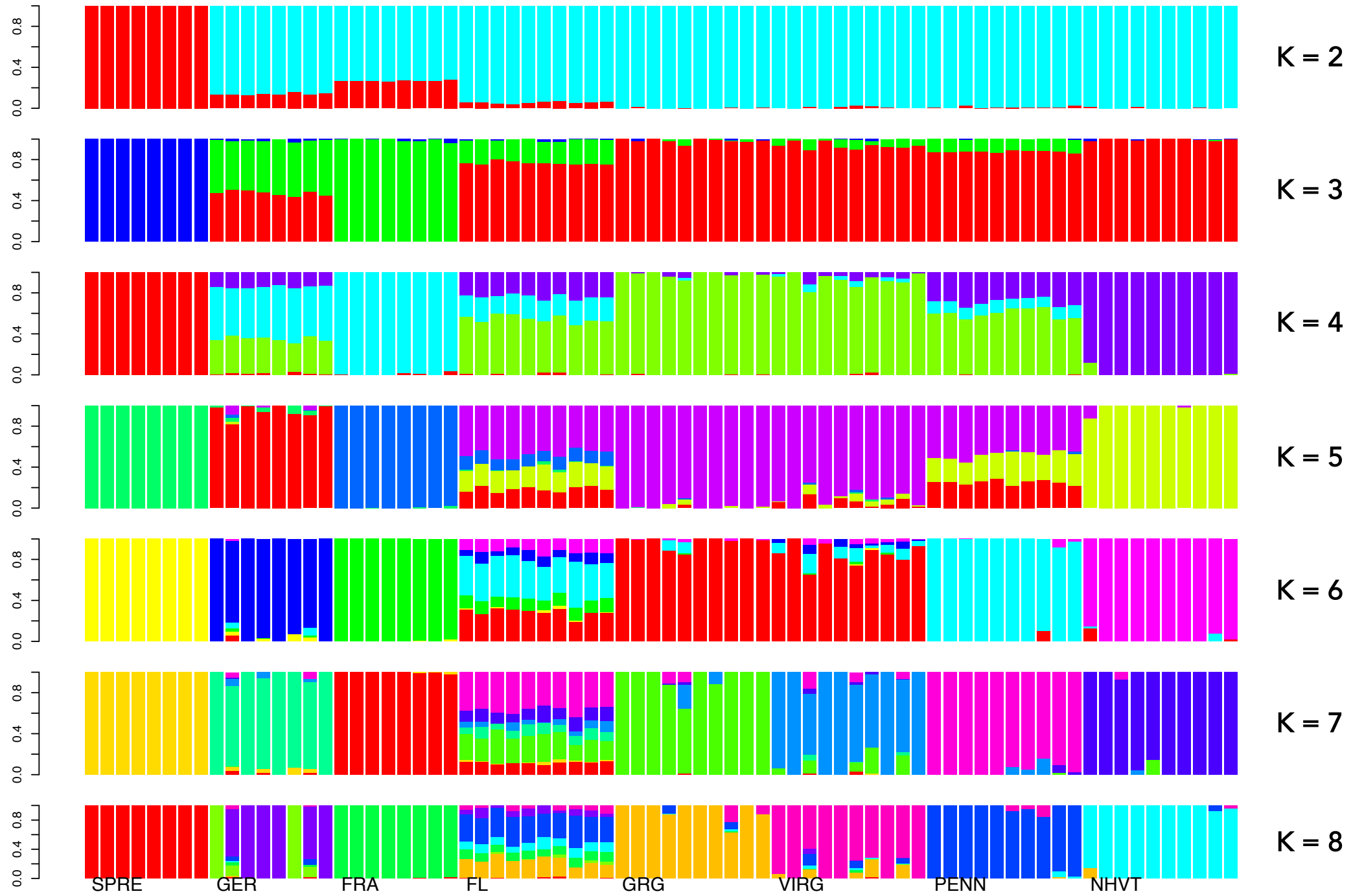

Supplement: jkac332_Supplementary_Data [file jkac332_supplementary_data.zip › Supplementary_Figure_1_G3-2022-403878.pdf]

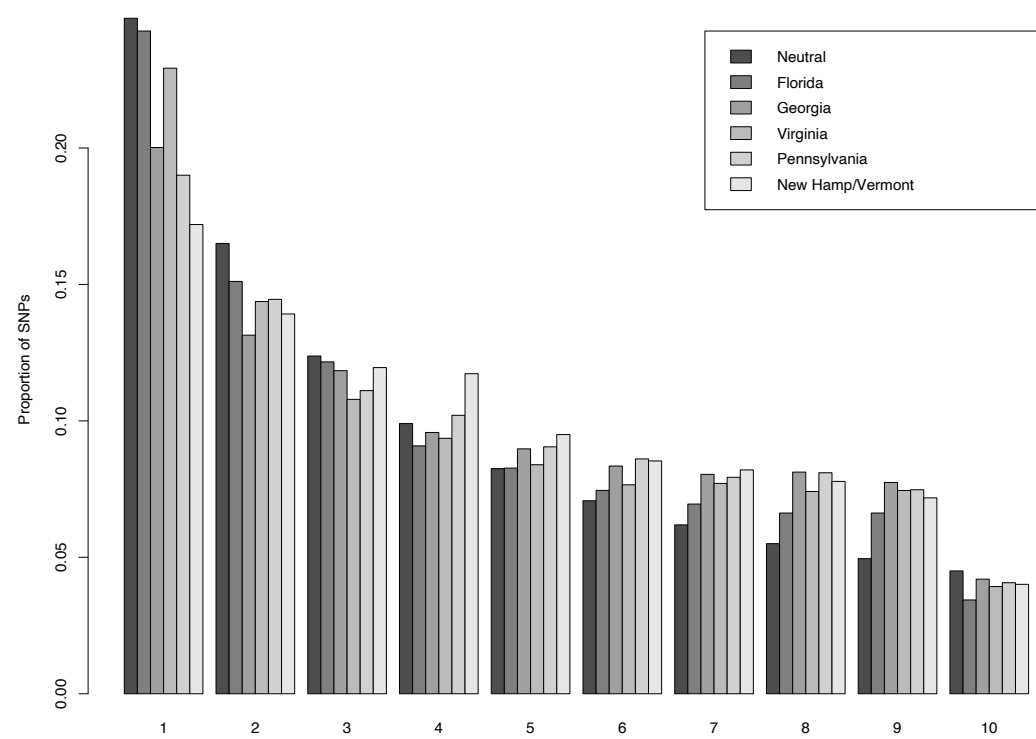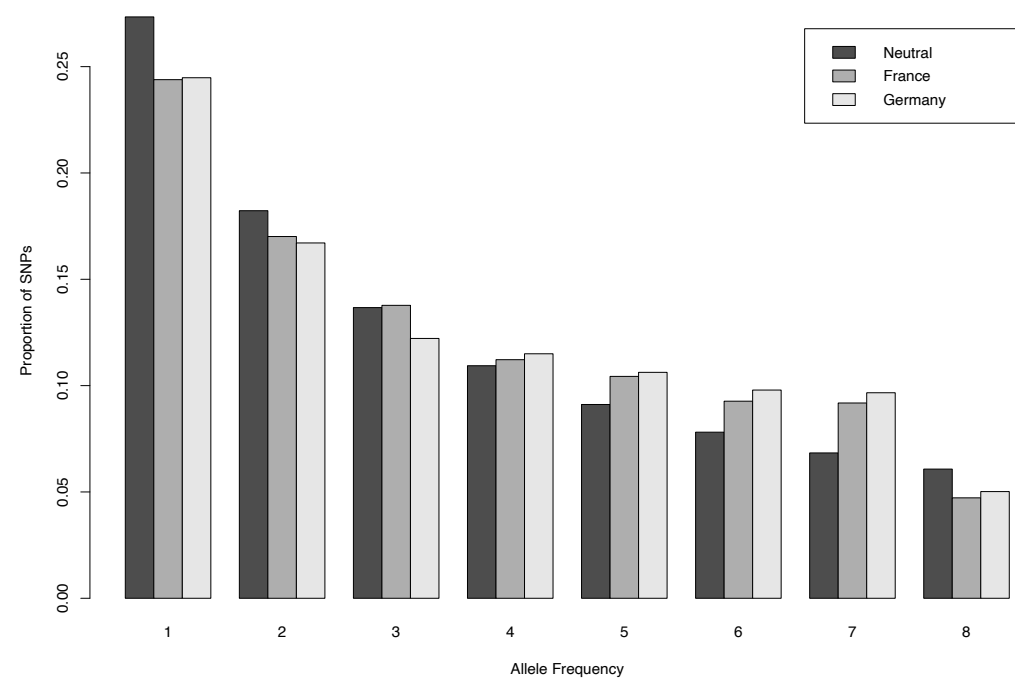

Supplement: jkac332_Supplementary_Data [file jkac332_supplementary_data.zip › Supplementary_Figure_2_G3-2022-403878.pdf]

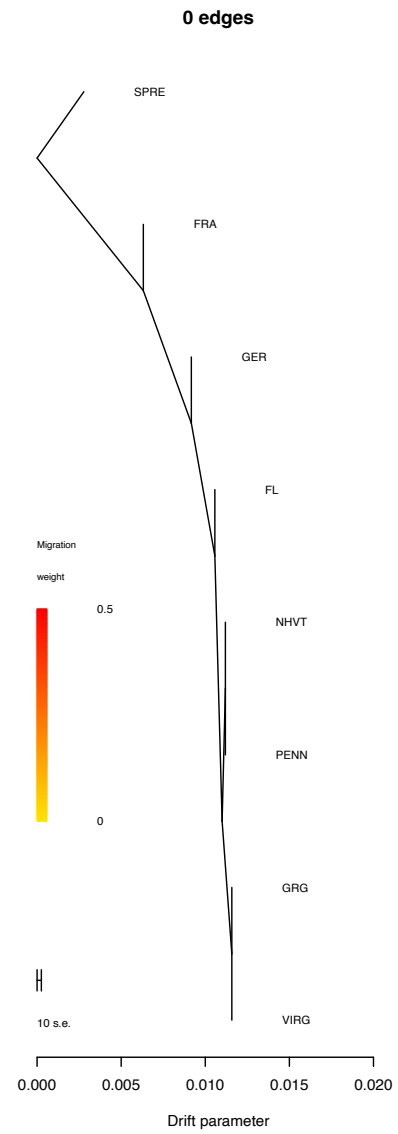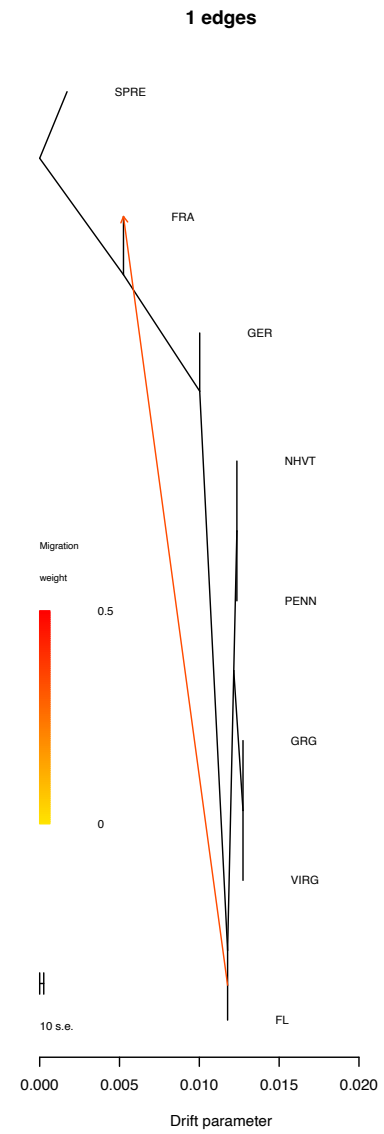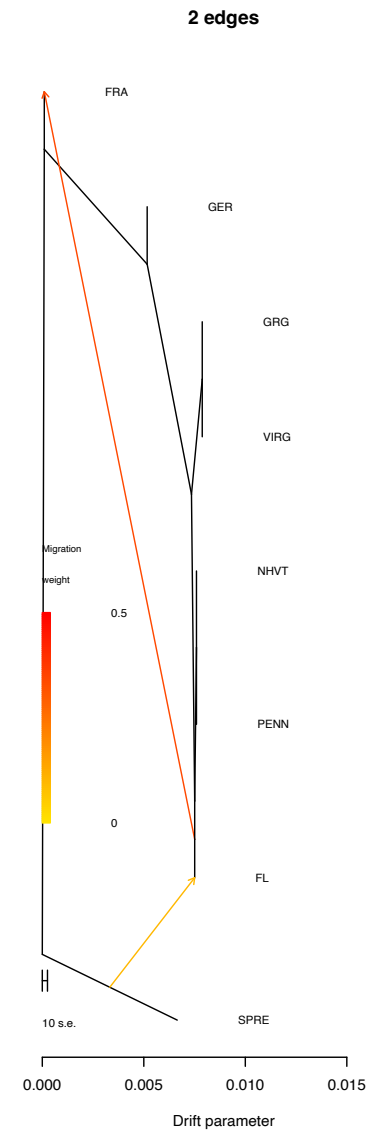

Supplement: jkac332_Supplementary_Data [file jkac332_supplementary_data.zip › Supplementary_Figure_3_G3-2022-403878.pdf]
